# Genito-Urinary Surgery

**Published:** 1905-06-03

**Authors:** 


### PROGRESS IN MEDICINE AND SURGERY.

### GENITO-URINARY SURGERY.

(*Continued from page* 157.)

**Venereal Warts.**—C. W. G. Rohrer (Baltimore)[3] discusses this condition. Age is no factor in the production of the warts. They are frequent in children, and in pregnant women suffering from irritating discharges. In either sex they may be met with in the urethra and inferior part of the rectum. Oberländer has described papillomatous urethritis. Most often the portion of the urethra affected is the navicular fossa or behind strictures. The author draws the following conclusions:—(1) The warts are hypertrophies rather than tumours, and result from irritation and inflammation; (2) 60 per cent. are venereal; 40 per cent. are non-venereal, and due to uncleanliness; (3) they are not confined to mankind, horses and dogs suffer from them; (4) when small, the best treatment is palliative; when large they are best removed surgically; even then recurrences are frequent; (5) the question as to their contagiousness is still *sub judice*; (6) numerous bacteria are embedded in their epithelium. The following remedies are mentioned:—Dusting with calomel, or with calomel one part and bismuth subnitrate two parts, or with powdered boric acid. A 10 per cent. salicylic acid ointment has advocates, or an ointment of resorcin gr. xv., vaseline ℥i. Valentine recommends touching them three times daily with ferric chloride. Powdered savin is said to be a specific (Posner). For surgical treatment a rat-toothed forceps and curved scissors are the best instruments, and a cocaine solution, 10 per cent., is an efficient anæsthetic.

**Torsion of the Spermatic Cord.**—J. Lacy Firth[9] reports a case of this affection and discusses the whole subject. The facts of interest in the case were as follows:—A man of 23, with the testis (right) completely descended, but possessing the anatomical peculiarity of being suspended freely in a horizontal position from the top of the tunica vaginalis by a pedicle of mesorchium, was attacked rather suddenly with severe pain in this testicle, followed in a few hours by swelling of the organ and œdema and redness of the scrotum, and on the thirty-second day the cause of the trouble was found by operation to be torsion of the spermatic cord and gangrene of the testicle. There are two forms of torsion of the cord—viz., one in which the testicle, together with the tunica vaginalis, is rotated, the cord being twisted outside the tunic, and one in which the cord is twisted inside the tunic. The former variety is comparable to the operative bistournage carried out in certain animals. Only three examples have been recorded. The second form is much commoner. The usual effect of the torsion on the testes is the production of hæmorrhagic infarction, the veins being more completely obstructed than the arteries. If the torsion is greater, and both the arteries and veins are completely obstructed, anæmic necrobiosis occurs, without any septic gangrenous processes. The essential cause of torsion appears to be the presence of a more or less elongated mesorchium, which suspends the testicle freely in the tunica vaginalis. The testicle may be fully or imperfectly descended. Exciting causes are such as lead to sudden and forcible contraction of the abdominal muscles, but there may be no ascertainable exciting cause. The age of the patients has varied from four months to sixty-two years. The symptoms are local and distant. Locally there is sudden pain in the testicle and iliac regions, followed by swelling of the testicle. Vomiting, pallor, sweating, and a rapid pulse are common. In one group of cases the symptoms quickly subside, but the patients have been liable to recurrences. The attacks may begin in sleep. The diagnosis has to be made chiefly from strangulated inguinal hernia if the testicle is in the groin, and from acute orchitis if the testicle is fully descended. The treatment may be detorsion by taxis, detorsion through an incision, detorsion with fixation of the testes in the scrotum to prevent recurrences, and orchidectomy, immediate or secondary.

**Undescended Testicle.**—Odiorine and Simmons (Boston)[10] contribute an article on this subject, based on a study of seventy-seven cases. The anomalies in the descent of testes may be considered in two groups, viz. (1) that in which the testis is arrested at some point in the normal course of descent (undescended testes), and (2) that in which the organ in its descent has deviated from the normal course (ectopia testis). The varieties of the former are (1) abdominal or iliac, when the testicle is in the abdomen; (2) inguinal, when it is in the inguinal canal; (3) pubic, when it is just outside the external ring; and (4) pubo-scrotal, when it is in the extreme upper part of the scrotum. The varieties of ectopia are: (1) perineal; (2) crural; and (3) peno-pubic. In the great majority of cases the "undescended" testis is incapable of forming spermatozoa, but not always. It has been maintained that in one group of cases the function of spermatogenesis is established for a few years, but is lost again early. The writers record one example in which a double cryptorchid, with marked atrophy of both testicles, was fertile at the age of 20, but apparently became sterile soon after that. The proportion of cases in which the undescended organ can form spermatozoa is greater than has been supposed. A person with one testicle fully descended is in no way affected as to bodily development or the power of procreation. The appropriate treatment of undescended testicle, without other complications such as hernia or inflammation, varies with the age of the patient. If a testicle is undescended at birth, there is still great probability that it will descend during the first few months of life and no treatment is required. In later childhood the longer the incomplete descent persists the smaller becomes the chance of spontaneous descent, and after 11 or 12 years of age this chance is very small. It is at the period of life from 11 or 12 years of age to puberty that operations for transplanting the testicle to the scrotum are most successful. If the testicle can be brought into the scrotum by manipulations these should be applied daily. Orchidopexy is the operation of choice in children, and in adults it should be attempted in most cases before orchidectomy is resorted to, but if the undescended testicle in adults is much atrophied and the other testicle is normal orchidectomy is the best procedure. In double arrest of descent orchidectomy should only be a last resort and castration is never justifiable. No treatment is advised for abdominal retention. A testicle replaced into the abdomen invariably becomes functionless, therefore both organs should never be returned there in children. The authors performed 24 orchidopexies, seven of these were in children ranging from five to 13 years of age. In only one case, a bilateral one, was the result perfect. Eleven operations were performed on adults from 16 to 42 years of age. In five cases the testicles remained in the scrotum though in a high position, except in one instance. Three testicles soon retracted into the inguinal canals, and two into the pubic region.

[8] Amer. Jour. Med. Sci., November, 1904, p. 761. [9] Bristol Medico-Chir. Jour., December, 1904, p. 320. [10] Annals of Surg., December, 1904, p. 962.

